# ﻿A new phylogeny of *Rumex* (Polygonaceae) adds evolutionary context to the diversity of reproductive systems present in the genus

**DOI:** 10.3897/phytokeys.204.85256

**Published:** 2022-08-05

**Authors:** Kirstie D. Grant, Daniel Koenemann, Janet Mansaray, Aisha Ahmed, Hamid Khamar, Jalal El Oualidi, Janelle M. Burke

**Affiliations:** 1 Department of Biological Sciences, Florida Agricultural & Mechanical University, Tallahassee, FL, USA; 2 Department of Biology, Howard University, Washington, DC, USA; 3 Department of Biological Sciences, Louisiana State University, Baton Rouge, LA, USA; 4 College of Medicine, Howard University, Washington, DC, USA; 5 Département de Botanique et Ecologie Végétale, Institut Scientifique, Université Mohammed V de Rabat, Rabat, Morocco; 6 Université Ibn Tofail, Faculté des Sciences, Laboratoire de Botanique et de Protection des plantes, B.P. 133, Kénitra, Morocco

**Keywords:** Dioecious, *Emex*, heteromorphic sex chromosome systems, monoecious, synoecious

## Abstract

*Rumex* is one of about 50 genera in the knotweed family, Polygonaceae. The genus comprises about 200 species with bisexual, or more commonly, unisexual flowers, with the species displaying monoecious, dioecious, synoecious (hermaphroditic) or polygamous reproductive systems. Some of the dioecious species have heteromorphic sex chromosomes, which is rare amongst angiosperms. We here present a plastid phylogeny of 67 species, representing all four subgenera. For this study, we used three chloroplast markers, *rbcL*, *trnH*-*psbA*, *trnL*-*F* and dense taxon sampling to reconstruct the most comprehensive molecular phylogeny of *Rumex* to date. The reconstructed phylogeny for this work resolves six major clades and one large grade in Rumexsubg.Rumex. In addition, the species with known dioecious reproductive systems are resolved within a broader clade we term “the dioecious clade”. These results suggest that the species with divergent reproductive systems are more closely related to each other than to other species comprising the rest of the *Rumex* genus.

## ﻿Introduction

Commonly known as docks and sorrels, *Rumex* L. (Polygonaceae) is a relatively large genus. *Rumex* encompasses four circumscribed subgenera, approximately 200 species and hundreds of described subspecies or varieties. Many species in *Rumex* are cosmopolitan in nature, spanning six continents of the world. However, many individual species are either regionally endemic, native or introduced on particular continents (Rechinger 1937).

**Figure 1.**
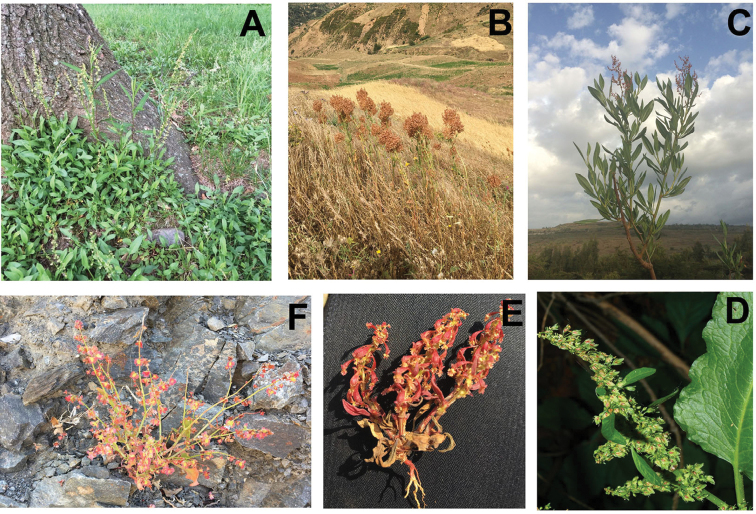
Depiction of morphological variation amongst the different subgenera of *Rumex*. **A***Rumexacetosella* growing in Virginia, USA (subg. Acetosella) **B***Rumexthyrsoides* growing in Morocco (subg. Acetosa) **C***Rumexnervosus* growing in Ethiopia (subg. Acetosa) **D***Rumexobtusifolius* growing in New York, USA (subg. Rumex) **E***Rumexbucephalophorus* collected on the Moroccan coast (subg. Platypodium) **F***Rumexpapilio* growing in Morocco (subg. Acetosa). All photo credits J.M. Burke.

The cosmopolitan distribution of *Rumex* species is indicative of their ability to thrive in a wide variety of environmental conditions. Described species are just as recurrent in dry and sandy soils as they are in marshes and cultivated fields, spanning the arctic, subarctic, boreal, temperate, tropical and subtropical localities (Löve and Kapoor 1967). Although several biological species demonstrate little to no niche preference (e.g. *Rumexcrispus* L., *Rumexobtusifolius* L.), there are others that exhibit exceedingly precise ecological requirements (e.g. *Rumexbipinnatus* L.f., *Rumexpictus* Forrsk.). The large variation in the distribution of *Rumex* species might also account for the large deviation observed in the morphology of these species (Fig. 1), whereby some reach almost seven metres in height and others rarely exceed a few centimetres (Rechinger 1949; Löve and Kapoor 1967; Rechinger 1990).

In the 20^th^ Century, progress in the taxonomic and cytological study of *Rumex* was largely accomplished by two researchers: Áskell Löve and Karl Heinz Rechinger (Rechinger 1937; Rechinger 1954a; Löve and Kapoor 1967). Löve extensively documented the cytological diversity of *Rumex* and he proposed a generic status for *Acetosa* and *Acetosella* (the groups with species bearing heteromorphic sex chromosomes) and subgeneric status for *Axillares* and *Platypodium*. Löve also considered *Rumex* to be composed of several smaller genera corresponding to a number of cytotypes (Löve 1957; Löve and Kapoor 1967; Mariotti et al. 2006, 2009).

Over the course of his career, Rechinger effectively monographed *Rumex*, using plant morphology and geographic distribution in his taxonomic treatments (Rechinger 1933, 1937, 1939, 1949, 1954a, b, 1984, 1990; Brandbyge and Rechinger 1989). By the mid-1900s, Rechinger had proposed a subgeneric status for *Platypodium* and maintained *Acetosa*, *Acetosella* and *Lapathum* as comparable subgenera (Rechinger 1954a; Table 1). Rechinger chose to retain *Rumex* as a single genus.

**Table 1.** Summary of the recognised subgenera in Rumex, with species diversity and reproductive systems present.

Subgenus	No. of species	Sexual system	Sex chromosomes
*Acetosa*	41	Dioecious, Gynodioecious, Polygamous	Yes (in part)- XX/XY_1_Y_2_
*Acetosella*	5	Dioecious (rarely polygamous)	Yes- XX/XY
*Rumex* (= *Lapathum*)	126	Synoecious, Monoecious	No
*Platypodium*	1	Synoecious	No

The reproductive systems of *Rumex* species vary extensively. Species of *Rumex* exhibit synoecious (hermaphroditic), monoecious, dioecious and polygamous reproductive systems (Rechinger 1949; Rechinger 1954a; Löve and Kapoor 1967; Mosyakin 2005; Navajas-Pérez et al. 2005). Most of the reproductive system diversity has been described in subgenera *Acetosa* or *Acetosella*. In particular, most species in these subgenera are dioecious (Rechinger 1937, 1949, 1954a, 1984). A few species in subgenus Rumex have variable systems, especially between synoecy and monoecy (e.g. *Rumexcrispus*, J. Burke, pers. obs.). There are also three species of *Rumex* endemic to the Hawaiian Islands (*Rumexalbescens* Hillebr., *R.giganteus* Aiton and *R.skottsbergii* O. Deg. & I. Deg.), which are all monoecious (Wagner et al. 1999).

*Rumex* has two different sex chromosome systems exhibited in many of the dioecious species, classified in Rumexsubg.Acetosa and Rumexsubg.Acetosella. In *Rumex*, the documented sex chromosomes are heteromorphic. Two sex-determining chromosomal mechanisms are known: XX/XY and XX/XY_1_Y_2_ (Löve 1940, 1942,1943, 1944; Löve and Löve 1948; Shibata et al. 1999, 2000; Navajas-Pérez et al. 2005; Cunado et al. 2007; Ming et al. 2011). The XX/XY_1_Y_2_ system is dosage-dependent and plant sex is based on the autosome to sex-chromosome ratio. In this system, female individuals have 14 chromosomes and male individuals have 15 chromosomes (Löve 1940, 1944; Löve and Kapoor1967; Navajas-Pérez et al. 2005).

Recent molecular phylogenetic work has sought to resolve the placement of *Rumex* in the Polygonaceae more broadly (Sanchez and Kron 2008; Sanchez et al. 2009; Burke et al. 2010; Burke and Sanchez 2011; Sanchez et al. 2011; Schuster et al. 2011, 2013, 2015). These studies have placed *Rumex* alongside the other Rumices of Campderá (*Emex* and *Oxyria*), with the addition of *Rheum* as either sister to *Oxyria* (Burke et al. 2010; Schuster et al. 2011) or to *Rumex* + *Emex* (Schuster et al. 2013, 2015). One area that lacks clarity has been the placement of *Emex*, which sometimes appears to be nested within *Rumex* (e.g. Sanchez et al. 2011) and is sometimes placed as sister to *Rumex* (e.g. Burke et al. 2010). Moreover, the relationships of species within *Rumex*, including the relationship between *Rumex* and *Emex*, continue to be poorly understood due to insufficient sampling and paucity of data. To date, the relationships amongst species placed within Rechinger’s subgenus Rumex are particularly obscure.

Here we present a new phylogeny of *Rumex*, constructed using three plastid gene regions (*trnH-psbA*, *rbcL* and *trnL*-*F*) and 67 *Rumex* species. We have used this phylogeny to test the placement and monophyly of its circumscribed subgenera, as well as discuss the broad patterns in the evolution of reproductive systems within *Rumex*.

## ﻿Materials and methods

### ﻿Taxon sampling and DNA Isolation

DNA was isolated from 109 accessions, representing 67 *Rumex* species. Of the 109 included accessions, a total of 99 *Rumex* accessions, six *Rheum* L. species, three *Emex* L. accessions and one species of *Persicaria* L. (Mill.) are represented. *Persicariavirginiana* (L.) Gaertn., *Rheumalexandrae* Batalin, *Rheumemodii* Wall., *Rheumnobile* Hook. f. & Thomson, *Rheumofficinale* Baill., *Rheumpalmatum* L. and *Rheumrhabarbarum* L. were included as outgroup species. Additional plant samples were obtained through the GenBank sequence database (Appendix A1). Samples were taken from a combination of herbarium specimens (K, NY, OSC, RAB, US), field collections and cultivated samples from collaborators. Herbarium acronyms follow the Index Herbariorum (Thiers 2019).

All fresh leaf samples were dried using silica gel. Plant tissue was homogenised using the FastPrep-24 5G Sample Preparation System (M. P. Biomedicals, LLC Santa Ana CA, USA). Total genomic DNA was extracted from herbarium specimen-sampled and silica-dried leaf tissues using a BIOLINE ISOLATE II Plant DNA Kit (Cat No. BIO-52070). Modification for herbarium material proceeded as follows: Cell lysis was carried out using 300 µl of buffer (PA1 or PA2) and 30 µl of proteinase K (20 µg/ml) and incubated for 18 hours at 65 °C on an orbital shaker.

### ﻿Marker selection

For this first comprehensive phylogeny of the genus, we focused on plastid marker selection. Previous authors utilised nrITS as a nuclear marker (Schuster et al. 2011; Schuster et al. 2015). However, we did not utilise nrITS for this phylogeny due to a number of issues that would interfere with accurate reconstruction of evolutionary relationships: 1) nrITS is extremely variable and difficult to align (66% of nrITS sequence data was excluded in Schuster et al. [2015] publication) and 2) Due to widespread polyploidy documented in multiple *Rumex* species, sequences of nrITS would not necessarily be low copy and there would be substantial issues with paralogy and orthology across multiple polyploidy events.

For plastid marker selection, we screened multiple markers that had previously been used in Polygonaceae reconstruction (Burke et al. 2010; Burke and Sanchez 2011; Koenemann and Burke 2020). We selected markers that both showed sufficient variation across the genus and were easily amplified for most taxa.

### ﻿PCR amplification and sequencing

Amplification of DNA markers was completed for three plastid regions: *rbcL*, *trnH-psbA* and *trnL-F* (Table 2). *rbcL* was amplified using the following PCR conditions: 94 °C for 1 min, followed by 34 cycles of 94 °C/15 s, 54 °C/15 s and 72 °C/30 s and a final extension period of 5 min at 72 °C. *trnH-psbA* was amplified using the following PCR conditions: 94 °C for 2 min, followed by 34 cycles of 94 °C/30 s, 55 °C/30 s and 72 °C/30 s and a final extension period of 7 min at 72 °C. *trnL-F* was amplified using the following PCR conditions: 80 °C for 5 min, followed by 34 cycles of 94 °C/1 min, 55 °C/1 min and 72 °C/2 min and a final extension period of 5 min at 72 °C. PCR and gel electrophoresis were performed following standard protocols with no special conditions. PCR experiments were performed separately with only fresh or only herbarium material to help prevent cross-contamination.

**Table 2.** Gene regions used: name of primers, total length of region, % parsimony informative characters.

Gene region	Reference	Primer names	Total aligned length	PIC (%)
*rbcL*	Fazekas et al 2008	rbcLF, rbcLR	539	24 (4.5)
*trnH*-*psbA*	Shaw 2007	psbA, trnH	596	132 (22.1)
*3trnL-F*	Shaw 2005	3’trnL^UAA^F, trnF ^GAA^	442	65 (14.7)
Combined			1577	221 (14.0)

PCR amplicons were sent to Eurofins Genomics (Louisville, KY) for Sanger sequencing. Sequences were edited using Geneious v. 10 (Biomatters Ltd.). Reviewed sequences were aligned with MUSCLE (Edgar 2004) and concatenated using MESQUITE (Maddison 2005).

### ﻿Phylogeny reconstruction

All phylogenetic analyses were completed using the CIPRES Science Gateway v. 3.3 (Miller et al. 2010). Prior to the phylogenetic reconstructions, we performed ModelTest-NG (Darriba et al. 2020) for the concatenated matrix to determine the suggested model of evolution. ModelTest-NG indicated that the best fit was the General Time Reversible (GTR) model.

We performed Maximum Likelihood (ML) phylogeny reconstruction using GARLI v. 2.01.1067 (Zwickl 2006). We used the default GARLI parameters with the following exceptions: we performed 1000 search replications (10 iterations of 100 search replicates). In order to better search tree space, we increased the attachments per taxon setting to 150 and extended the generations without improvement parameter to 50000. To evaluate support for phylogenetic relationships, statistical bootstrapping was performed, specifying only one search replicate per bootstrap iteration for 100 iterations. All bootstrap trees were downloaded and used to generate a majority rule consensus tree in MESQUITE (Maddison 2005). The consensus tree was visualised in FigTree version 1.4.3 (Rambaut 2014).

We performed Bayesian Inference phylogeny reconstruction in MrBayes 3.2.7a (Ronquist et al. 2012). The priors were set to the defaults (Dirichlet). We set the seed number at 123. We conducted two independent Markov Chain Monte Carlo (MCMC) runs, each with four chains employing BEAGLE library acceleration (as recommended by CIPRES). Each MCMC run was set to complete 5 million generations, with trees sampled every 1,000 generations. The first 25% of trees in each run were discarded as burn-in. MrBayes then synthesised the two independent runs and extracted the majority rule consensus tree with posterior probabilities.

Posterior probability and bootstrap values were visualised using FigTree version 1.4.3 (Rambaut 2014) and MESQUITE (Maddison 2005). Posterior probabilities above 90% and bootstrap support values above 70% were considered significant and annotated in the final phylogeny.

## ﻿Results

The most likely tree was generated using 109 specimen accessions. This included seven outgroup species, three accessions of *Emex* and 99 accessions of *Rumex*. The present phylogeny represents 67 *Rumex* species, more than twice the number of species of *Rumex* sampled in previous phylogenies (31 species in Navajas-Pérez et al. 2005; 13 species in Schuster et al. 2015). A total of 47 sequences were missing from the final matrix, yielding 14.4% missing data in the final analysis (Grant 2022). Table 2 summarises the variability of each of the gene regions. The most variable region was *trnH-psbA*, which consisted of 22.1% parsimony informative characters. The least variable region was *rbcL* which consisted of 4.5% parsimony informative characters.

The most likely tree recovered by GARLI received a likelihood score of Ln = -5767.548440.

**Figure 2.**
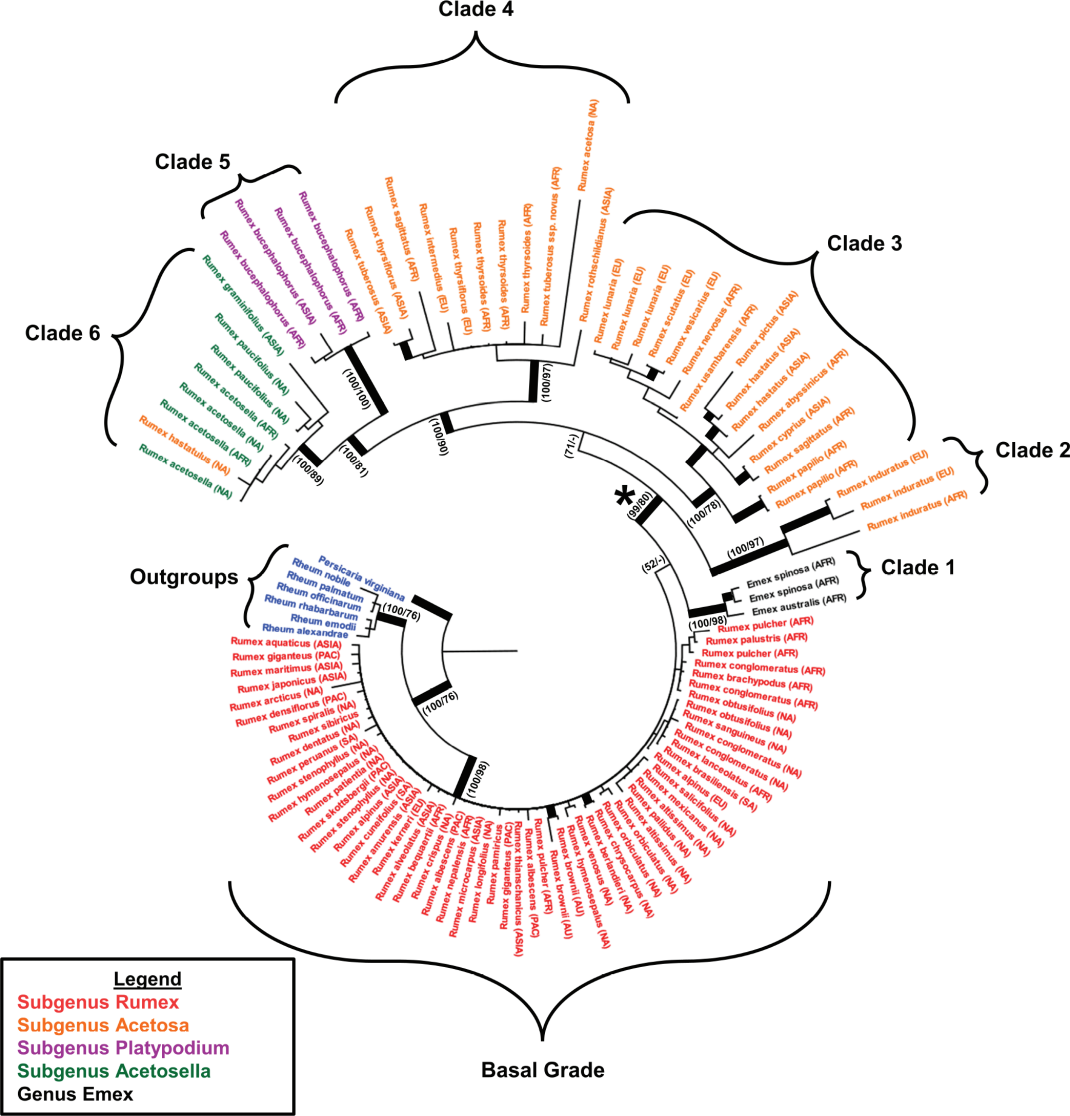
Bayesian phylogenetic reconstruction for *Rumex* species using three chloroplast sequences (*rbcL*, *trnH*-*psbA* and *trnL*-*F*). Thickened branch indicates simultaneous posterior probability above 90% and bootstrap support above 70%. Exact support values are indicated at important nodes (Bayesian Posterior Probability/Maximum Likelihood Bootstrap). Outgroup species (*Rheum* and *Persicaria*) are shown in blue. Rumex species, traditionally placed in subgenus Rumex, are shown in red. Species, traditionally placed in the sister genus *Emex*, are shown in black. Rumex species, traditionally placed in subgenus Acetosa, are shown in gold. Rumex species, traditionally placed in subgenus Platypodium, are shown in purple. Rumex species, traditionally placed in subgenus Acetosella, are shown in green. The asterisk denotes the transition to dioecy “Dioecious Clade” referenced in the text.

The genus *Rumex* was recovered as monophyletic with strong support (100 Bayesian Posterior Probability/98 Maximum Likelihood Bootstrap) (Fig. 2). The analysis did not recover RumexsubgenusRumex, the subgenus with the most species diversity, as monophyletic. In our phylogeny, species of subgenus Rumex form a grade at the base of the tree (“Basal Grade” – Fig. 2). *Emex* was recovered as monophyletic, just above the Basal Grade and sister to the dioecious clade. While the results indicate strong support for the relationship between the known *Emex* species, *E.australis* and *E.spinosa* (100/98), they are conflicting and show poor support for the placement of *Emex* within *Rumex*. Posterior probability support for the placement of *Emex* is only 52% and the most likely GARLI tree placed Emex within the Basal Grade of subgenus Rumex. Furthermore, different gene regions reconstructed conflicting topologies for the placement of *Emex*. The *rbcL* phlyogeny placed *Emex* within RumexsubgenusRumex (50% bootstrap support). Both *trnh-psbA* and *trnL*-*F* placed *Emex* as sister to the *Rumex* genus (*trnh-psbA* < 50% bootstrap support and *trnL*-*F* 91% bootstrap support) (results not shown).

The remaining taxa, comprising the subgenera *Acetosa*, *Acetosella* and *Platypodium* form a highly supported (99/80) monophyletic group (Fig. 2). This group is denoted as “the dioecious clade” because it is here that we see the initial transition to dioecy of the known dioecious *Rumex* species resolved in this group. The relationships of the clades within this group are also well-supported. Our recovered phylogenetic tree did not recover subgenus Acetosa as monophyletic. Within the dioecious clade, subgenus Acetosa is comprised of three well-supported, monophyletic groups, Clade 2 (100/97), Clade 3 (100/78) and Clade 4 (100/97) and is nested below a pair of clades, represented by subgenus Platypodium (Clade 5) and subgenus Acetosella (Clade 6). The pair (*Platypodium* + *Acetosella*) is also well supported (100/81). Subgenus Platypoidium was recovered as monophyletic with strong support (100/100) and consists of four accessions of its only circumscribed species: *Rumexbucephalophorus*. Species in subgenus Acetosella were recovered together with strong support (100/89), but the inclusion of *Rumexhastatulus* means the subgenus was not recovered as monophyletic.

In addition to corresponding largely to the established subgeneric system, the topology also largely corresponds to the diversity of the reproductive and sex chromosome systems present in *Rumex*. Species in subgenus Rumex (Basal Grade) are mostly hermaphroditic with no documented heteromorphic sex chromosomes. With no documented heteromorphic sex chromosomes, *Emex* is also represented as a clade and consists of purely monoecious species. Subgenus Acetosa consists entirely of dioecious species, with some members exhibiting the sex chromosome system XX/XY_1_Y_2_. Subgenus Platypodium, another hermaphroditic group with no reported sex chromosomes, is nested between subgenera *Acetosa* and *Acetosella*. Subgenus Acetosella consists of species that are both dioecious and have the sex chromosome system XX/XY.

## ﻿Discussion

Our results produced a phylogeny of *Rumex*, with six major clades and one grade, largely congruent with Rechinger’s subgeneric classification. The placement of *Emex* conflicted, based on the molecular markers used. In our phylogeny, it is sister to the dioecious clade, but without strong support.

Within the phylogeny, the basal grade is mostly made up of species from RumexsubgenusRumex. That subgenus Rumex was recovered as a grade rather than a clade is not surprising given the known extensive hybridisation amongst species of this subgenus. This phenomenon most certainly contributed to the lack of resolution in species-level relationships within subgenus Rumex. Additionally, although hybridisation between species in subgenus Rumex and species in the other subgenera are not well documented, it is possible that such hybrids exist and serve to hinder our ability to distinguish subgenus Rumex as a clade. We suspect that increased taxon sampling and genetic data, especially from the nuclear genome, will help to resolve relationships amongst species in subgenus Rumex.

Although dioecious, the species included in Clade 2 and Clade 3 have no reported heteromorphic sex chromosome systems. The species included in Clade 4 exhibit a complex sex chromosome system (XX/XY_1_Y_2_). This placement suggests that this heteromorphic sex chromosome system was derived from dioecious ancestors. The genetic origin of heteromorphic sex chromosomes in *Rumex* is beyond the scope of this manuscript, but this result provides a framework to investigate potentially intermediary taxa that may contain homomorphic or transitionary sex chromosome systems.

Subgenus Platypodium (Clade 5) was resolved as monophyletic and nested within “the dioecious clade”. Based on its plant and chromosome morphology, earlier studies concerning *Rumexbucephalophorus* have referred to it as the link between subgenus Rumex, which is predominantly synoecious and subgenus Acetosella, which is predominantly dioecious (Löve 1944). Although morphologically variable, *R.bucephalophorus* consistently exhibits a synoecious reproductive system. Its derivation from amongst the dioecious species in this phylogeny suggests a reversal from a dioecious condition.

Subgenus Acetosella (Clade 6) was not recovered as monophyletic. Known dioecious species, *R.hastatulus*, of subgenus Acetosa is nested within subgenus Acetosella. *Rumexhastatulus* is documented to exhibit two distinct karyotypes: a complex sex chromosome system (XX/XY_1_Y_2_, North Carolina karyotype) which is characteristic of subgenus Acetosa and the simple sex chromosome system (XX/XY, Texas karyotype) which is characteristic of subgenus Acetosella (Navajas-Pérez et al. 2005; Mariotti et al. 2009; Hough et al. 2014). In addition, Rechinger’s 1937 treatment indicates a polygamous reproductive system for *R.hastatulus* (Rechinger 1937). Given the variability found within this species, *R.hastatulus* could have been placed in either subgenus (*Acetosa* or *Acetosella*), where species appear to have diversified according to the type of sex chromosome system they exhibit. This finding suggests the plasticity of reproductive and sex chromosome systems within *Rumex*, as a single species can exhibit two different karyotypes.

One of the striking features of the phylogeny recovered in this study is its congruence with the taxonomic system established by Rechinger (Rechinger 1933, 1937, 1939, 1949, 1954a, 1954b, 1984, 1990). Rechinger retained the diversity of species as a single genus, but divided them into four subgenera: Rumex (Lapathum), *Platypodium*, *Acetosa* and *Acetosella*. Each subgenus is prominently present in the topology. Subgenus Platypodium is monophyletic. Subgenus Acetosella is monophyletic even with the inclusion of *Rumexhastatulus*, whose placement has been ambiguous. The two largest subgenera, *Acetosa* and *Rumex*, were recovered as grades. The grade of subgenus Acetosa is well-resolved and well-supported. The grade of subgenus Rumex is both less well-resolved and less well-supported. The recovered topology, nevertheless, serves to confirm the major relationships amongst species in the genus, relationships for which Rechinger had proposed using only morphology.

In all, this work has provided a reconstructed phylogeny that differs from those currently published (Navajas-Pérez et al. 2005; Schuster et al. 2015) and has tested the placement and monophyly of its circumscribed subgenera. This work builds on those previous studies by providing an increased taxon sampling density, which has resulted in a more comprehensive reconstruction of the evolutionary history of *Rumex* and a more thorough examination of the stability of the subgeneric system. This work has provided an early outline of the evolution of reproductive systems in *Rumex*, suggesting an ordered plasticity and transitions from synoecy to dioecy to dioecy with heteromorphic sex chromosomes. Additionally, this work suggests a possible reversal from a dioecious condition. Future directions in *Rumex* research include the identification and application of nuclear markers that will allow for a more robust phylogeny, particularly with respect to the placement of *Emex*. Additionally, future genomic studies will serve to elucidate the evolution of the sex chromosomes and sex determining regions in *Rumex*.

## ﻿Appendix A1. List of taxa sampled and vouchers specimens

Abbreviations of herbaria where the voucher is housed are listed after the collection number. Sequences can be found on the Dryad database: https://doi.org/10.5061/dryad.69p8cz8zs

For sequences that we did not generate, accession information is given as found on GenBank.

**GenBank sequences used for this study**:

*rbcL*: *Rumexpamiricus* Rech. f. – JF944139.1, *Rumexsibiricus* Hulten- KC483892.1

*trnH-psbA*: *Rumexpamiricus*- JN047053.1

**Table A1.** DNA Sequences Generated for this Study.

Scientific name	Voucher
*Emexaustralis* Steinh.	P.C. Zietsma 4053, NY
*Emexspinosa* (L.) Campd.	Schuhwerk 90/328, NY
*Emexspinosa* (L.) Campd.	J.M. Burke 302, HUDC
*Persicariavirginiana* (L.) Gaertn.	J.M. Burke s.n., BH
*Rheumalexandrae* Batalin	Cultivated Material, HUDC
*Rheumemodii* Wall.	Cultivated Material, HUDC
*Rheumofficinale* Baill.	Cultivated Material, HUDC
Rheumpalmatumvar.taguticaum L.	Cultivated Material, HUDC
*Rheumrhabarbarum* L.	Cultivated Material, HUDC
*Rheumnobile* Hook. f. & Thomson	Pradham 820581, BH
*Rumexabyssinicus* Jacq.	J.M. Burke 251, HUDC
*Rumexacetosa* L.	K.D. Grant s.n., HUDC
*Rumexacetosella* L.	R. Brand 1336, NY
*Rumexacetosella* L.	D.E. Atha 10521, NY
*Rumexacetosella* L.	K.D. Grant s.n., HUDC
*Rumexacetosella* L.	J.M. Burke 309, HUDC
*Rumexalbescens* Hillebr.	Lorence 5224, K
*Rumexalbescens* Hillebr.	Wood 14959, US
*Rumexalpinus* L.	Larsen 20708, US
*Rumexalpinus* L.	D.E. Atha 5114, NY
*Rumexaltissimus* Alph, Wood	Shultz 8717, US
*Rumexaltissimus* Alph. Wood	D.E. Atha 10857, NY
*Rumexalveolatus* Los.-Losinsk.	Rechinger 48318, US
*Rumexamurensis* F. Schmidt ex Maxim.	Barrett Lilan22p
*Rumexaquaticus* L.	Elias 7251, US
*Rumexarcticus* Trautv.	Shetler 4560, US
*Rumexarifolius* All.	K. Deguchi 4023, NY
*Rumexbequaertii* De Wild.	Germishuizen 3447, US
*Rumexberlandieri* Meisn.	Thieret 17178, US
*Rumexbrachypodus* Rech. f.	J.M. Burke 312, HUDC
*Rumexbrasiliensis* Link	R. Wasum 1655, NY
*Rumexbrownii* Campd.	Wilson 10250, NY
*Rumexbrownii* Campd.	Wilson 10250, US
*Rumexbucephalophorus* L.	Barrett 17RBTA5
*Rumexbucephalophorus* L.	J.M. Burke 293, HUDC
*Rumexbucephalophorus* L.	J.M. Burke 301, HUDC
*Rumexbucephalophorus* L.	J.M. Burke 304, HUDC
*Rumexchrysocarpus* Moris	D.E. Atha 13012, NY
*Rumexconglomeratus* Murray	D.E. Atha 10045, NY
*Rumexconglomeratus* Murray	J.M. Burke 271, HUDC
*Rumexconglomeratus* Murray	J.M. Burke 298, HUDC
*Rumexconglomeratus* Murray	J.M. Burke 299, HUDC
*Rumexcrispus* L.	J.M. Burke 268, HUDC
*Rumexcuneifolius* Campd.	J.C. Solomon 13044, US
*Rumexcyprius* Murb.	Kocher B-273, US
*Rumexdensiflorus* Osterh.	Pinkava P12626, US
*Rumexdentatus* L.	D.G. Kelch 07.328, OSC
*Rumexgiganteus* Aiton	K. Thorne 6736, NY
*Rumexgiganteus* Aiton	Canfield 1304, US
*Rumexgraminifolius* Gerogi ex Lamb.	Petrosky 1811, US
*Rumexhastatulus* Baldwin	D.E. Atha 10503, NY
*Rumexhastatus* D. Don	MacArthur 1291, US
*Rumexhastatus* D. Don	Barrett s.n.
*Rumexhymenosepalus* Torr.	Cultivated material, HUDC
*Rumexhymenosepalus* Torr.	A. Tiehm 15727, OSC
*Rumexinduratus* Bioss. et Reut.	M.W. Chase 925, K
*Rumexinduratus* Bioss. et Reut.	Barrett s.n.
*Rumexinduratus* Bioss. et Reut.	J.M. Burke 310, HUDC
*Rumexintermedius* DC.	Rainha 5270, US
*Rumexjaponicus* Houtt.	Bai-Zhang 4049, US
*Rumexkerneri* Borbás	Barta 2004-390, US
*Rumexlanceolatus* Thunb.	H.J. Venter 10295, NY
*Rumexlongifolius* DC.	D. E. Atha 8858, NY
*Rumexlunaria* L.	NR. 8879, NY
*Rumexlunaria* L.	Barrett 17RLLM1
*Rumexlunaria* L.	Barrett 17RLTF1
*Rumexmaritimus* L.	Shiu Ying Hu 13127, US
*Rumexmexicanus* Meisn.	D.E. Breedlove 13305, US
*Rumexmicrocarpus* Campd.	Barrett MJ-P40 (Seed)
*Rumexnepalensis* Spreng.	J.M. Burke 248, HUDC
*Rumexnervosus* Vahl	J.M. Burke 252, HUDC
*Rumexobtusifolius* L.	J.M. Burke s.n., BH
*Rumexobtusifolius* L.	J.M. Burke 270, HUDC
*Rumexorbiculatus* A. Gray	Ruee 43716, US
*Rumexorbiculatus* A. Gray	D.E. Atha et al. 8883/2010, NY
*Rumexpallidus* Bigelow	D.E. Atha 13922, NY
*Rumexpalustris* Sm.	J.M. Burke 306, HUDC
*Rumexpapilio* Coss. & Balansa,	S.L. Jury 13659, K
*Rumexpapilio* Coss. & Balansa	J.M. Burke 303, HUDC
*Rumexpatientia* L.	D.E. Atha 10674, NY
*Rumexpaucifolius* Nutt.	Barrett 17RpCOT3.2
*Rumexpaucifolius* Nutt.	Barrett 17RpCMC15.2
*Rumexperuanus* Rech. f.	V. Quipuscoa 1349, NY
*Rumexpictus* Forssk.	Barrett 17Rp.AR1
*Rumexpulcher* L.	J.M. Burke 294, HUDC
*Rumexpulcher* L.	J.M. Burke 295, HUDC
*Rumexpulcher* L.	J.M. Burke 296, HUDC
*Rumexrothschildianus* Aarons. ex Evenari	Barrett 17Rrs3.2
*Rumexsagittatus* Thunb.	Strobach B55575, US
*Rumexsagittatus* Thunb.	H.J. Venter 9995, NY
*Rumexsalicifolius* Weinm.	W. Wood s.n., OSC
*Rumexsanguineus* L.	J.M. Burke 316., HUDC
*Rumexscutatus* L.	Barrett s.n.
*Rumexskottsbergii* O.Deg. & I.Deg.	Degener 35050, US
*Rumexspiralis* Small.	D.E. Atha 9727, NY
*Rumexstenophyllus* Ledeb.	D.E. Atha 11389, NY
*Rumexstenophyllus* Ledeb.	R.L. McGregor 40643, OSC
*Rumextianschanicus* Losinsk.	Barrett SH1-A-2007454
*Rumexthyrsiflorus* Fingerh.	Ollegard 261, US
*Rumexthyrsiflorus* Fingerh.	Elias 7282, US
*Rumexthyrsoides* Desf.	J.M. Burke 305, HUDC
*Rumexthyrsoides* Desf.	J.M. Burke 313, HUDC
*Rumexthyrsoides* Desf.	J.M. Burke 307, HUDC
*Rumextuberosus* L.	S. Omar et al. 52591, K
*Rumextuberosus* subsp. nov	J.M. Burke 308, HUDC
*Rumexusambarensis* (Dammer) Dammer	Ellemann 889, NY
*Rumexvenosus* Pursh.	R.E. Brainerd 428, OSC
*Rumexvesicarius* L.	Brummit 15271, US

## Supplementary materials

Supplementary material 1 Aligned Data Matrix Data type FASTA Explanation note Aligned sequence file. https://binary.pensoft.net/file/724137This dataset is made available under the Open Database License (http://opendatacommons.org/licenses/odbl/1.0/). The Open Database License (ODbL) is a license agreement intended to allow users to freely share, modify, and use this Dataset while maintaining this same freedom for others, provided that the original source and author(s) are credited.Kirstie D. Grant
